# Prostatic vascular damage induced by cigarette smoking as a risk factor for recovery after holmium laser enucleation of the prostate (HoLEP)

**DOI:** 10.18632/oncotarget.12538

**Published:** 2016-10-09

**Authors:** Huan Xu, Chong Liu, Meng Gu, Yanbo Chen, Zhikang Cai, Qi Chen, Zhong Wang

**Affiliations:** ^1^ Department of Urology, Shanghai 9th People's Hospital, Shanghai Jiaotong University School of Medicine, Shanghai, China

**Keywords:** benign prostatic hyperplasia, holmium laser enucleation of the prostate, smoking, vascular damage, perioperative characteristics

## Abstract

**Purpose:**

To evaluate the relationship between prostatic vessel changes induced by cigarette smoking and the perioperative outcome of holmium laser enucleation of the prostate (HoLEP).

**Materials and Methods:**

A total of 268 postoperative patients with benign prostatic hyperplasia (BPH) were prospectively analysed in our department. They were divided into two groups (smokers and non-smokers) according to smoking history. Transrectal colour Doppler ultrasound was performed to evaluate the prostate vascular changes. Pathologically, HE staining, CD31 and CD34 were analysed in prostatic section chips. Furthermore, postoperative outcomes were determined during a 6-month follow-up period.

**Results:**

The preoperative prostate volume was significantly decreased in smoking patients (*P* = 0.04). CPI was significantly lower in smoking BPH patients (*P* < 0.01), whereas RI was significantly increased in smokers compared with non-smokers (*P* < 0.01). Histological assays revealed elevated CD34 in the smoking BPH individuals presenting an increased number of microvessels. The HoLEP duration was increased in smokers. Interestingly, we identified significantly increased overactive bladder syndrome score (OABSS) and decreased Qmax in smoking individuals during the 6-month follow-up with no difference being observed preoperatively. However, no significant difference between the groups was observed for the International Prostate Symptom Score (IPSS).

**Conclusions:**

The significantly lower CPI and higher RI values in smoking BPH patients indicated the presence of considerable vascular damage in these subjects. Moreover, cigarette smoking extended the surgical duration and prolonged the recovery period of overactive bladder (OAB) syndrome. Thus, integrated treatment should be suggested for various BPH individuals.

## INTRODUCTION

Benign prostatic hyperplasia (BPH), one of the most common proliferative disorders in older males, is characterized by increasing tissue mass in prostatic transition zone. The pathogenic process of BPH involves various factors, including chronic inflammation, oxidative stress, hypoxia and ischaemia [1, 2]. These pathogenic factors may result in generalized or localized vessel disorder, which play significant role in BPH [3]. In a vicious circle, vascular damage also results in ischemia and hypoxia, which aggravates the disease progression [4, 5]. Although some researchers have argued that hypoxia and ischemia may accelerate cell proliferation and vascular damage may contribute to BPH [3], the anti-apoptotic effect is dependent on the severity and duration of anoxia [6, 7]. Thus, prostatic vascularity has always been a focus of studies about prostate enlargement.

Surgery is currently the most efficient treatment for BPH. Surgical methods have developed from an open method to transurethral endoscopic surgery. There is a growing emphasis on reducing the risk of surgery and accelerating postoperative recovery. However, local vessel changes in the prostate make performing surgery difficult and delay postoperative recovery. Although there have been numerous studies in the area of ischemia and BPH, little is known regarding the relationship between vessel damage and postoperative recovery.

Colour Doppler ultrasound (CDUS) is a non-invasive technology used to study blood flow. Computer-assisted quantification of CDUS by the calculation of colour pixel intensity (CPI) has been shown to be an accurate method to assess organ and tissue perfusion [8]. Pulsed-wave Doppler ultrasound is utilized to obtain the resistive index (RI), which represents both blood flow and pressure. RI represents one of the most relevant indicators of vascular injury in the evaluation of small prostatic vessels [9]. Pathologically, microvessel density (MVD) is identified using immunohistochemical staining for CD31 and CD34 [8, 10]. CD34 is expressed in pericytes of blood vessels standing mainly for the existing vessels, whereas CD31 is a pan-endothelial cell marker associated with newly formed microvessels [10].

It is widely accepted that cigarette smoking induces chronic inflammation, hypoxia and endothelial injury [11–13]. Interestingly, numerous studies have reported that cigarette smoking reduces prostate volume, and some studies have even claimed that smoking delays BPH [14–16], although other researchers held the opposite views in their own studies [17, 18]. However, the mechanism by which smoking induces prostatic vascular changes remains unknown. Furthermore, with surgery being the most efficient treatment for BPH, there is still no evidence for the relationship among chronic vascular disorders, smoking and perioperative characteristics.

The present study examined the relationship between cigarette smoking and prostatic vascular damage in BPH patients using contrast-enhanced CDUS and immunohistochemistry. We also evaluated the perioperative and follow-up data for smoking patients.

## RESULTS

**Figure 1:**
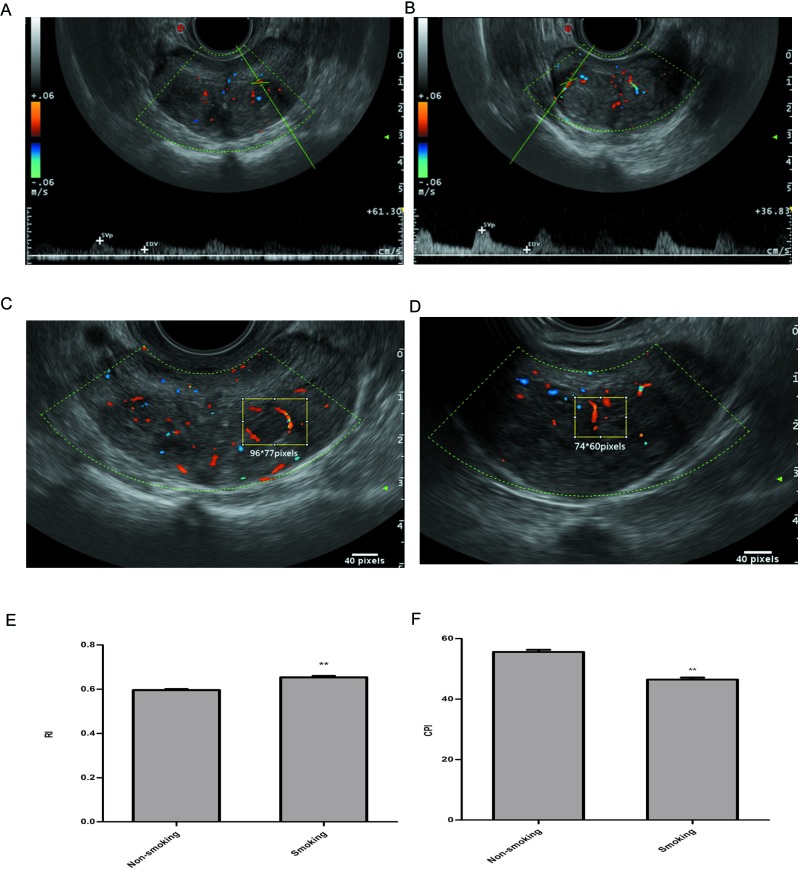
Representative images and analysis of RI and CPI in transrectal colour Doppler ultrasound on prostatic blood flow **A**. RI for TZ vascular bundles of non-smokers, **B**. RI for TZ vascular bundles of smokers, **C**. CPI measurements of non-smokers, **D**. CPI measurements of smokers **E**. TZ vascular RI comparison between smokers and non-smokers; **F**. vascular CPI comparison between smokers and non-smokers; P* < 0.05, P** < 0.01.

Data were obtained from 268 patients who underwent HoLEP during a 1-year period. The baseline characteristics of patients are listed in Table 1. A slightly decreased prostate volume was observed in smoking patients (*P* = 0.04). The blood metabolic tests revealed that testosterone was significantly elevated in the smoking group (*P* < 0.01), whereas there were no significant differences in the other aspects, including serum PSA levels. CDUS data were collected preoperatively and divided into non-smoking vs. smoking groups according to the criteria. Interestingly, the vessel RI was significantly enhanced in the smoking group compared with the non-smoking group (*P* < 0.01). The results of the CPI are shown in Figure 1. It was significantly decreased in smoking patients (*P* < 0.01).

**Table 1:** Baseline pre- and post- operative characteristics

	Mean ± SD (range) Non-smoking	Mean ± SD (range) Smoking	*P*-value
Pt. age (y)	72.53±7.28	71.50±8.67	0.29
BMI	23.37± 2.17	23.29±2.15	0.76
PV (ml)	61.07±18.75	56.63±14.56	0.04*
PSA (ng/ml)	1.96±1.11	1.78±1.04	0.22
BG (mmol/l)	6.07±6.66	5.34±1.29	0.22
TG (mmol/l)	1.04±0.49	1.07±0.43	0.62
CHO (mmol/l)	3.85±0.63	3.95±0.75	0.25
HDL (mmol/l)	1.09 ±0.17	1.13±0.18	0.09
LDL (mmol/l)	2.37±0.50	2.36±0.53	0.81
FFA (mmol/l)	0.36±0.15	0.36±0.15	0.50
Testosterone (ng/ml)	3.81±1.42	5.54±1.80	<0.01**
Haemoglobin decrease (g/dl)	1.16±0.65	1.19±0.42	0.63
Resected weight (g)	48.32±6.25	46.56±6.23	0.95
Serum sodium decrease (mmol/L)	3.53±0.74	3.49±0.85	0.69
Operative time (h)	81.13±11.51	84.63±13.42	0.04*
Catheterization time (d)	3.69±1.06	3.77±1.13	0.58
Hospital stay (d)	4.55±1.12	4.58±1.32	0.87

PV: prostate volume; BG: blood glucose; TG: triglyceride; CHO: cholesterol;

HDL: high-density lipoprotein; LDL: low-density lipoprotein; FFA: free fatty acids;

*P* *<0.05, *P* **<0.01

**Figure 2:**
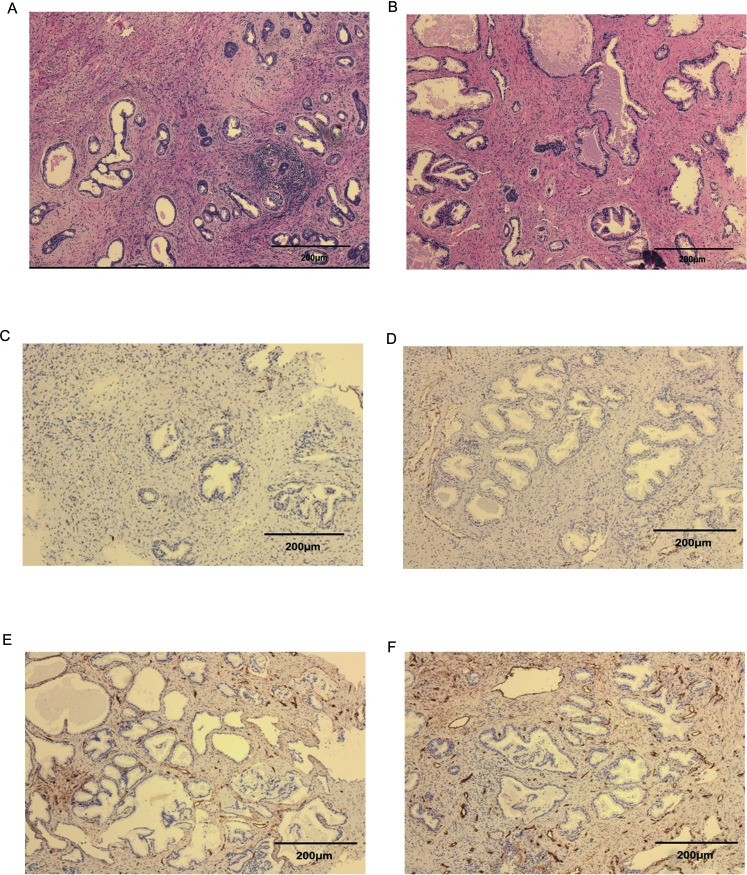
HE staining and immunohistochemical evaluation of prostatic tissue and brown-stained spots representing vessels at 100× magnification **A**. HE for sections of non-smokers and **B**. sections of smokers. **C**. newly formed vessels (CD31) of non-smokers, **D**. newly formed vessels (CD31) of smokers, **E**. lower MVD (CD34) of non-smokers, and **F**. higher MVD (CD34) of smokers.

The results of HE staining of the postoperative sections are displayed in Figure 2 demonstrating the presence of pathological prostatic hyperplasia in both groups. No obvious differences were observed between the two groups. Moreover, patients with prostatic cancer were excluded from our study according to the pathological analysis. The resected prostate chips were histologically examined for CD31 and CD34, ensuring variation of MVD. Although no significant differences in CD31 staining were observed, the smoking group exhibited stronger CD34 staining, suggesting an elevated MVD in this group (shown in Figure 2).

**Figure 3:**
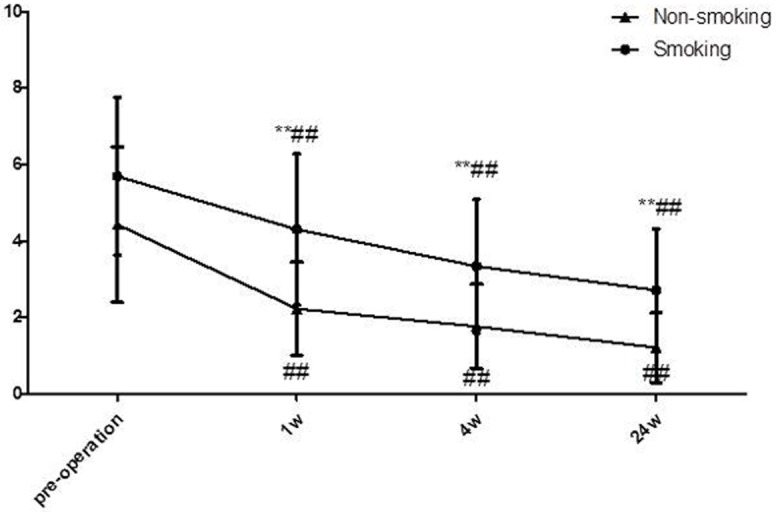
Preoperative and postoperative OABSS curves at the 1-w, 4-w and 24-w follow-ups P* < 0.05, P** < 0.01, compared with the pre-operation; *P*^#^ < 0.05, *P*^##^ < 0.01, non-smoking group compared with smoking group.

The perioperative parameters were recorded and are listed in Table 1. These parameters were similar in the two groups, except for a longer surgical duration (*P* = 0.04) in the smoking group. At the 6-month follow-up, the two groups did not significantly differ with respect to IPSS. Interestingly, the analysis of OABSS revealed a higher score for the smokers compared with the non-smokers in terms of 1-, 4- and 24-week postoperative data, although the preoperative scores of the two groups were similar. The variation curve of OABSS was shown in Figure 3. The Qmax and urinary volume were lower in the smoking group at the 4-week follow-up, whereas no differences between the groups were observed in the preoperative and 24-week follow-up data (shown in Table 2).

**Table 2:** Follow-up data

	Baseline	1 w	4 w	24 w
Mean ± SD IPSS				
Non-smoking	20.55±4.72	11.82±4.34	10.10±3.98	6.10±2.05
Smoking	20.87±5.03	12.70±3.74	11.11±3.68	6.39±2.23
P-value	0.64	0.12	0.06	0.35
Mean ± SD OABSS				
Non-smoking	5.32±2.06	2.23±1.22	1.77±1.10	1.21±0.91
Smoking	5.70±2.07	4.31±1.97**	3.35±1.75**	2.72±1.20**
P-value	0.19	<0.01	<0.01	<0.01
Mean ± SD Qmax (ml/s)				
Non-smoking	7.57±3.68	—	24.22±3.57	24.70±3.61
Smoking	7.84±3.73	—	21.69±4.69**	23.94±4.45
P-value	0.58	—	<0.01	0.24
Mean ± SD volume (ml)				
Non-smoking	197.17±29.81	—	232.22±28.02	261.02±27.93
Smoking	192.23±33.88	—	199.58±21.64**	255.20±26.07
P-value	0.23	—	<0.01	0.18

*P* *<0.05, *P* **<0.01

Complications were reported during the 6-month follow-up period. One patient in the non-smoking group required recatheterization after catheter removal due to urine retention, whereas none of the patients in the smoking group required recatheterization. One non-smoking patient (vs. none in the smoking group) required a blood transfusion and reoperation for haemostasis. Various degrees of urinary incontinence were present in the postoperative patients, but this was resolved within 1 month after surgery. Posterior urethral strictures occurred in two patients and were resolved by internal urethrotomy. Bladder neck sclerosis was not observed in our study.

## DISCUSSION

As is shown in Table 3, some urologists explored the relationship between smoking and BPH [14, 16, 19–30]. Some investigations have evaluated the relationship between smoking and BPH, demonstrating either a moderate inverse association between smoking and BPH or no association [14, 15, 26, 31, 32]. Other studies have reported a protective effect of smoking on BPH and prostatism [33]. In addition, the degree of cigarette smoking was associated with the persistence of the effects on the prostate: light or moderate smokers were less likely to have moderate to severe prostatism and BPH, whereas heavy smokers tended to have moderate to severe prostatism and BPH compared with non-smokers [16, 17]. Our results presented that the prostate volume was decreased in smokers compared with non-smokers (61.07±18.75 ml vs. 56.63±14.56 ml, *P* = 0.04). These findings suggest that cigarette smoking affects plasma steroid hormone levels, particularly by elevating testosterone concentrations. Higher plasma testosterone tends to be associated with higher intra-prostatic dihydrotestosterone (DHT) levels, which has been demonstrated to be important during the development of BPH [26, 34]. Moreover, nicotine has been shown to lead to DHT accumulation in the canine prostate and to increase sympathetic nervous system activity contributing to BPH and LUTS [26, 35]. In smokers with BPH, the cigarette tobacco composition and serum PH may also play a role in decreasing serum zinc levels, which has been reported to affect the amounts of both testosterone and DHT in the prostate [36]. Significantly elevated testosterone level was observed in our analysis, whereas no differences were observed in PSA levels or other metabolic factors. Thus, most studies have focused on the relationship between cigarette smoking and the pathological process of BPH. This report is the first study to investigate the effects of cigarette smoking on vascularity and perioperative characteristics.

**Table 3:** Previous studies on the relation between smoking and BPH

Author	Publication Year	Distinct	Age scope	Study design	BPH definition	N, cases	Smoking
Sidney S [28]	1991	Oakland or San Francisc	≥40	cohort	Surgery or symptmatic	16291	P
Seitter WR [27]	1992	California	40-79	cohort	Not mentioned	929	No
Chyou PH [12]	1993	Hawaiian island	>50	cohort	surgery	6,581	No
Roberts RO [14]	1994	Minnesota	40-79	cross-sectional	Sympotamatic and PV	2115	N
Matzkin H [23]	1996	Israel	41-97	prospective	Sympotamatic	195	N
Kupeli B [22]	1997	Turkey	52-57	Not mentioned	Surgery or symptmatic	280	P
Platz EA [25]	1998	Not mentioned	40-75	cohort	Surgery or symptmatic	29,386	P
Signorello LB [29]	1999	Greece	Not mentioned	Not mentioned	Not mentioned	430	No
Meigs JB [24]	2001	Boston	40-70	cohort	Surgery or symptmatic	1019	P
Gass R [20]	2002	Switzerland	65-80	cohort	Surgery or symptmatic	882	P
Kang D [21]	2004	International	55-74	cohort	Surgery or symptmatic	34,694	No
Rohrmann S [16]	2005	USA	≥60	cross-sectional	Sympotamatic	2797	No
Fritschi L [19]	2007	Western Australia	40–75	case–control	surgery	869	No
Sarma AV [26]	2010	Olmsted County	40-79	cohort	surgery	2089	No

P, positive association; N, negative association; No, no association;

BPH, which is caused by an increased number of cells in the prostatic TZ, is also characterized by a change in vascularization. As a previous study reported, vascular damage and prostate cell proliferation share a common pathogenic mechanism. The vessel RI is low in healthy patients and is advanced in BPH patients. Some studies have attributed this finding to compressed arteries between the peripheral zone (PZ) and the TZ, resulting in a marked increase in the RI of the capsular arteries[37]. Other studies investigating the vascular anatomy of the normal prostate have found notable differences between the RI values of the PZ and TZ. These studies have reported significantly higher RI and lower CPI induced by diabetes in TZ but not in PZ [3]. In this study, we only analysed the CPI and RI in the TZ. We found that RI was significantly elevated smoking group compared with non-smokers (0.59 ± 0.05 vs. 0.65 ± 0.06, *P* < 0.01). Furthermore, decreased prostate volume was observed in the smokers after excluding the elevated compression from the enlarged PZ. These findings indicated significant vascular damage with increased vascular resistance in the smoking patients. CPI, as an analysis of tissue perfusion, confirmed the results that vessel damage resulted in poor organ perfusion of the prostate. Although CD34 showed the presence of increased microvessels in the prostates of smokers, organ hypoperfusion may still exist.

**Figure 4:**
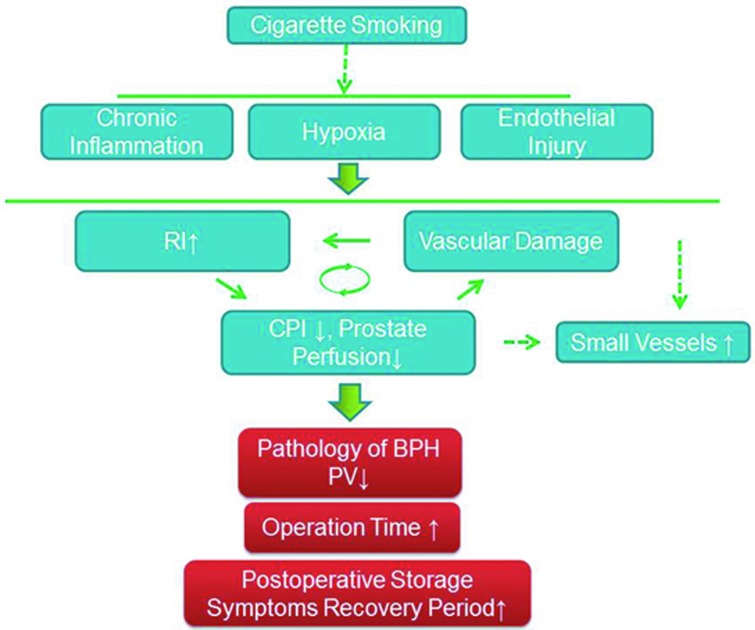
Schematic model of the effects of smoking on prostate vascular and perioperative characteristics The dashed arrow indicates the potential pathway of the effects of cigarette smoking. Bold arrows indicate indirect effects. Cigarette smoking tends to cause vascular injury via local hypoxia, endothelial injury and chronic inflammation in the prostate. The vessel damage, increased vascular RI and decreased prostate perfusion form a vicious circle. Moreover, these factors enhance the microvascular density. All of these factors play a role in the development of BPH and affect the perioperative characteristics.

The detailed mechanism underlying these findings had not previously been clarified. Cigarette smoking, as is widely accepted, has been demonstrated to cause vascular injury via local hypoxia, oxidative stress, endothelial injury and chronic inflammation in different organs [38–41]. In addition, vessel damage might cause the above adverse factors leading to a vicious circle. Moreover, damage of small vessels tends to result in organic or local ischemia. It has been reported that chronic prostatic vascular ischemia, hypoxia and chronic inflammation result in increased prostate volume[42]. However, these effects are dependent on the severity and duration. Severe ischemia triggered by the castration[43] of animals and the embolization of prostate vessels causes a reduced prostate[7, 44]. Elevated microvessel levels, as visualized by CD34, might be a compensatory effect, which cannot correct the hypoperfusion caused by the vascular damage. In addition, inflammatory factors and hypoxia have been shown to result in an increase in microvessels [45, 46] (Figure 4).

Perioperative characteristics and follow-up data were also obtained during our prospective research. No significance was investigated between the two groups, except the operative time (81.13±11.51 vs. 84.63±13.42 h, *P* = 0.04). The increased operative duration in smokers might also result from the prolonged morcellation duration caused by structural changes, including stromal fibrosis and endothelial growth[42]. Although we developed the morcellation technique as previously reported, more time was still spent on the treatment of prostatic tissue, which consisted of more fibrosis[47]. Moreover, proliferating microvessels made it difficult to do enucleation and stop haemostasis during surgery. Thus, cigarette smoking tended to not affect surgical difficulty, except for a slightly extended surgical duration.

Defined as a score of more than 7 points on the IPSS, LUTS is commonly observed in BPH patients. As previously reported, there is controversy about the relationship between chronic smoking and LUTS[26]. Some researchers have argued that heavy smokers have a higher risk of LUTS compared to moderate smokers [17]. Many researchers have attributed this finding to nicotine, which increases sympathetic nervous system activity, the tone of the smooth muscle of the bladder and prostate, and testosterone levels [26]. The OABSS was developed to assess OAB syndrome, which can be caused by an obstruction due to BPH; OABSS is a useful tool for research and clinical practice [48]. Thus, we examined LUTS and OABSS in the follow-up study. In our analysis, no significant difference in IPSS was observed between smoking and non-smoking patients pre- or post- operatively. Moreover, OABSS showed no difference between the two groups preoperatively, which was consistent with results obtained in previous reports. Interestingly, the 1-w, 4-w, and 24-w postoperative follow-up studies revealed a significantly elevated OABSS in the smoking patients. This finding may be partly correlated with the relief of obstruction after surgery. The main syndrome before surgery was obstruction, which may present as LUTS and mask the OAB syndrome. After the enlarged prostate was removed, OAB syndrome was prominent. As some previous studies have reported, smoking may partially enhance the contractile activity of the urinary bladder, resulting in a decrease in postoperative urinary retention [49]. Furthermore, Qmax was evaluated in the outpatient follow-up procedure. The Qmax in the smoking group was significantly lower compared with the non-smokers (24.22±3.57 ml/s vs. 21.69±4.69 ml/s, *P* < 0.01) at the 4-w follow-up. However, the urinary volume was lower in the smoking patients, which may have been caused by OAB syndrome, resulting in a lower Qmax.

A major limitation of this study is that it did not elucidate the precise mechanism underlying cigarette smoking and prostate development. In addition, this study does not further our understanding of the prevention and treatment of vascular damage in BPH patients.

In conclusion, this prospective study demonstrated significantly lower CPI and higher RI values for smokers compared with non-smokers in BPH subjects, indicating considerable vascular damage in smoking BPH patients. Moreover, cigarette smoking slightly prolonged the operative duration and delayed OAB syndrome recovery. Thus, we recommend that BPH patients quit smoking preoperatively to improve their vascular damage, and medicine to improve OAB syndrome should be administered to thesepatients after surgery. Further studies should focus on the potential of integrated clinical treatment to decrease urinary complications and increase patient satisfaction.

## MATERIALS AND METHODS

This prospective cohort comparison study has been performed at our medical centre from August 2015 to July 2016. In total, 268 patients treated at our hospital for lower urinary tract symptoms (LUTS) and obstruction due to BPH were included in the study. All of the patients underwent holmium laser enucleation of the prostate (HoLEP) due to urinary syndrome. Ethical approval was obtained, and written informed consent was obtained from the patients. Among the patients, 131 were non-smokers and 137 were cigarette smokers. The men who were enrolled in the study were asked to provide information, including age, weight, height, alcohol consumption, use of cigarettes, and medical history. The inclusion criteria were indications for the surgical treatment of BPH (eg, urinary retention, recurrent urinary tract infections, bladder stones or diverticula, treatment-resistant macroscopic haematuria, or dilatation of the upper urinary tract due to benign prostatic obstruction [BPO] with or without renal insufficiency)[50]. We excluded patients with severe pulmonary disease, heart disease, renal function impairment, any type of bleeding disorder, type 2 diabetes, hyperlipaemia, severe obesity (BMI ≥30 kg/m^2^), poor blood pressure control, alcohol consumption, neurogenic bladder dysfunction, bladder cancer, previous prostate surgery, PSA >4 ng/ml, prostate cancer, or urethral stricture. Smoking history was obtained during the interview, and the subjects were classified as smokers (patients who currently smoked more than 1 cigarette per day or had formerly smoked more than 100 cigarettes in their lifetime) or non-smokers (patients who had never smoked before). During the interview, patients with long-term alcohol consumption habits were excluded from the study. We categorized patients as drinkers if they drank alcohol more than once per week [26, 51].

Baseline characteristics consisting of urological history, concurrent diseases, previous drug therapy, International Prostate Symptom Score (IPSS), overactive bladder syndrome score (OABSS) and Qmax were collated prior to surgery. Preoperative blood tests, including PSA, serum sodium, haemoglobin and metabolic index, were also performed. All of the ultrasound investigations were performed before HoLEP by a single experienced radiologist and consisted of contrast-enhanced CDUS in which the RI was measured by pulsed-wave spectral Doppler analysis. Three to five waveforms of each of the participant's vessels were obtained, and the RIs from these waveforms were calculated to obtain the mean RI. The CPI values of transitional zones (TZs) were evaluated using computer-assisted quantification. We identified the region of interest as that with the highest detectable blood flow. ImageJ (1.46r; National Institutes of Health, USA) was utilized to post-process the output images. Details of the procedure that was followed were published by Berger, A. P. et al. in 2005 [3].

All the HoLEP procedures were performed by the same surgeon Zhong W. HoLEP was performed with a 550 m firing laser fibre and a 100 W continuous flow VersaPulse^®^ holmium laser from Lumenis. A 27 Fr resectoscope with a modified bridge was used to hold the laser fibre (Storz, Tuttlingen, Germany). The power settings were 80-100 W at 2-1.5 J per second and 50-40 Hz. Transurethral morcellation was performed through a 26 Fr nephroscope using a mechanical morcellator (VersaCut™).

Postoperatively, we immediately assessed the serum sodium and haemoglobin levels, the operative time, and the resected prostatic weight. Catheterization time and hospitalization duration were also noted. Epidural anaesthesia and the lithotomy position were applied during surgery. The irrigation fluid (normal saline) was hung 60 cm above the operating table. An irrigating catheter was inserted and bladder irrigation was performed until haematuria was resolved. Catheter removal was dependent on urine colour without gross haematuria. Follow-up data were collected 1 week, 4 weeks and 24 weeks after surgery by a single experienced doctor. The data consisted of IPSS, OABSS and late postoperative complications. The surgical method and perioperative treatment were published by our group in 2012 [52].

The resected prostate mass was fixed in 10% buffered formalin, embedded in paraffin and stained with haematoxylin and eosin for pathological analyses. Next, the tissue chips were histologically analysed for CD31 and CD34 staining. After 30-min antigen fixation in a microwave, a blocking solution (1% BSA + 0.1% Tween-20 in PBS) was added. The slides were incubated overnight with an anti-CD31 antibody (Abcam, ab28364, USA) at the recommended concentration of 1:50, as well as with anti-CD34 (Abcam, ab81289, USA) at a concentration of 1:100. After four washes in blocking solution, the slides were incubated with a secondary antibody for 30 minutes, and the nuclei were counterstained with haematoxylin. Areas with the most intense vascularization were evaluated by scanning at 100× magnification. For each patient, three to five images were collected and analysed. Immunofluorescence intensity was quantified using ImageJ (1.46r; National Institutes of Health, USA).

Based on our research expenses and the study period, CDUS was performed on 102 smokers and 95 non-smokers. Tissue masses were collected from at least 40 patients in each group. Preoperative tests were performed for at least 115 patients in both groups. Our follow-up collection of IPSS and OABSS data included 105 non-smokers and 107 smokers; 24 patients were lost to follow-up. Similar to the Qmax and urinary volume analysis, preoperative data were collected from 114 non-smokers and 123 smokers. At least 105 patients from each group were selected for immunohistochemistry and HE staining. Data were recorded at 4 and 24 weeks during outpatient visits. Measurement data were statistically analysed using a two-tailed Student's t-test and are presented as the means ± SD. ANOVA was used to assess the variance in IPSS and OABSS during the 24-week follow-up. All statistical analyses were performed using a commercially available statistical package (SPSS 19.0; IBM). A p-value ≤0.05 was considered statistically significant.
